# Intelligent Path-Selection-Aided Decoding of Polar Codes

**DOI:** 10.3390/e25020200

**Published:** 2023-01-19

**Authors:** Hongji Cui, Kai Niu, Shunfu Zhong

**Affiliations:** Key Laboratory of Universal Wireless Communications, Ministry of Education, Beijing University of Posts and Telecommunications, Beijing 100876, China

**Keywords:** polar codes, successive cancellation list, path selection, sort, neural network

## Abstract

CRC-aided successive cancellation list (CA-SCL) decoding is a powerful algorithm that dramatically improves the error performance of polar codes. Path selection is a major issue that affects the decoding latency of SCL decoders. Generally, path selection is implemented using a metric sorter, which causes its latency to increase as the list grows. In this paper, intelligent path selection (IPS) is proposed as an alternative to the traditional metric sorter. First, we found that in the path selection, only the most reliable paths need to be selected, and it is not necessary to completely sort all paths. Second, based on a neural network model, an intelligent path selection scheme is proposed, including a fully connected network construction, a threshold and a post-processing unit. Simulation results show that the proposed path-selection method can achieve comparable performance gain to the existing methods under SCL/CA-SCL decoding. Compared with the conventional methods, IPS has lower latency for medium and large list sizes. For the proposed hardware structure, IPS’s time complexity is $O(k\log_{2}\left(L\right))$ where *k* is the number of hidden layers of the network and *L* is the list size.

## 1. Introduction

Polar codes [1] were the first channel coding method to achieve channel capacity with a low-complexity encoder and decoder. The CA-SCL [2] decoding algorithm was proposed to improve the performance of polar codes compared to LDPC codes under finite code length.

SCL [3] and CA-SCL are the most popular decoding algorithms for polar codes. However, there are two challenges in improving the throughput of SCL decoding: (1) Data dependence of successive cancellation in the decoding processes; the decoder can only decode bit by bit. (2) Path selection: usually, a metric sorter is used for path selection.

In this paper, we focus on path selection. Path selection selects the best *L* candidate paths from all 2L candidate paths. Traditionally, the path metric (PM) is used for metric sorting, and *L* paths with the smallest path metric are selected as the surviving paths. In software simulation, metric sorting can be done using methods such as bubble sorting and quick sorting. In hardware implementation, it is necessary to consider the balance of hardware resources and latency.

The LLR-based path metric and pruned radix-2L sorting were proposed in [4]. The LLR-based PM has some good properties that can reduce the complexity of sorting. If *L* PMs are completely sorted, the 2L PMs after path extension are partially ordered, and there is no need to sort all the 2L PMs. Based on this property, pruned bitonic sorting and bubble sorting [5] were proposed, both of which can reduce hardware complexity.

Focusing on reducing hardware complexity and latency, double thresholding sorting [6], odd-even sorting [7], pairwise metric sorting [8], hybrid bucket sorting [9], and other methods have been proposed, one after another. It is worth noting that the research proposes a non-sorting direct selection strategy that uses bitwise comparison of PMs to select surviving paths and lowers resource complexity and latency [10]. However, all of the above methods come at a cost. Low resource consumption and delays will result in performance loss. When the list becomes larger (L>32), resource consumption and delay will increase sharply, which limits the throughput of the CA-SCL decoder. At the same time, for the polarization-adjusted convolutional (PAC) codes, the authors of [11] proposed a local sorting method that picks only the best path at each state node, thereby greatly reducing the sorting latency.

Permutation entropy [12] was proposed in 2002 to analyze the complexity of time series. Certainly, permutation entropy can be used to measure the chaotic degree of the PM sequence. By comparing the change in permutation entropy before and after extension, we can get a new measure of the complexity of path selection.

In recent years, neural networks have been applied to channel coding and decoding. In this paper, we exploit a neural network to perform path sorting and achieve a high-throughput SCL/CA-SCL decoder. The first neural sorting network was proposed in 1997 [13]. It requires the time complexity of O(1), but the latency is still huge for a CA-SCL decoder. In 2020, Tim Kraska proposed an ML-enhanced sorting algorithm called Learned Sort [14], which is based on learned index structures [15]. The core of the algorithm is to train the cumulative distribution function (CDF) model *F* over a small sample of data *A* to predict the position of test data *x*; the test set size is *M*: $pos=F_{A}\left(x\right)\cdot M=P\left(A\leq x\right)\cdot M$. In fact, it is impossible to train a perfect CDF model, so positional collisions will inevitably occur. Model establishment and collision handling methods will affect network performance and speed. This kind of neural network is usually used for processing big data, such as databases.

Inspired by neural network sorting methods, we have designed intelligent path selection (IPS) to replace the traditional path sorter. IPS is different from traditional sorters or neural sorting networks. Actually, IPS is not a sorter but a binary classifier. There is no need to sort from 1 to 2L because all paths have been divided into good paths and bad ones.

**Figure 1:**
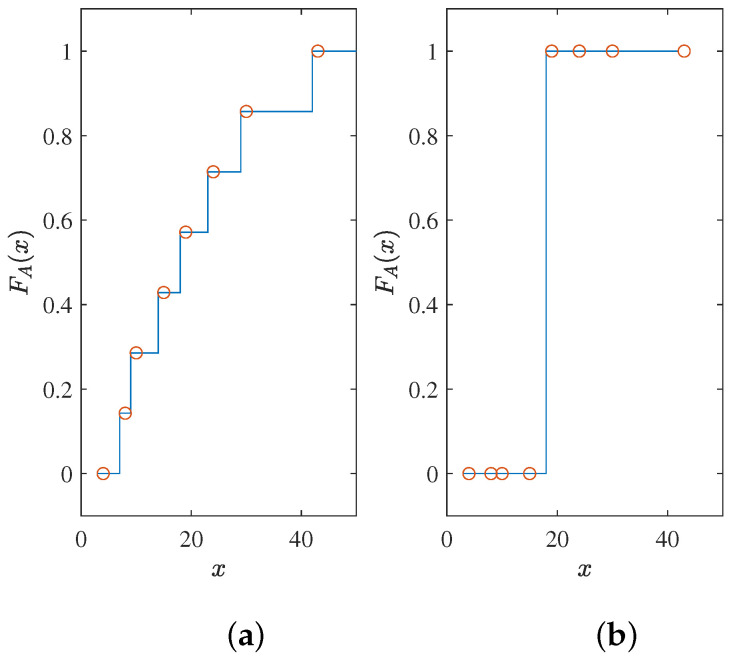
(**a**) Fully sorted CDF; (**b**) CDF of path selection of SCL.

Here, we give an example. For an unordered array *A* = [8, 10, 15, 24, 19, 4, 30, 43], the perfect CDF for its full sorting is a staircase function, and Figure 1a is one of them. However, for the path selection of SCL, it only needs to be a step function, and one of the possibilities is Figure 1b. The key to the problem is the position of the step. For a one-dimensional problem, it is an equivalent problem. If a suitable step position is obtained, the CDF is obtained. On the contrary, getting a CDF means that the suitable step position is known. For the path selection problem, a natural step position is the median. Knowing the median makes it easy to divide the data into two parts, larger and smaller. However, in SCL decoding, the arrays dynamically change, which is why we need a neural network to handle the problem. The neural network will give us a CDF or median that can adapt to dynamic arrays. In this study, we chose to train with a target output similar to the CDF.

In this way, the system’s complexity will be significantly reduced. Next, we designed a simple neural network for training: a universal neural network that can be adapted to different code lengths and code rates. Finally, we designed a threshold and a path selection matching strategy to match the network’s output and the SCL/CA-SCL decoder. The hardware structure of the fully connected neural network is designed to have highly parallel [16] performance to maintain overall lower latency, and the pipelined structure [17] reduces resource consumption and improves the utilization of hardware resources. The simulation results show that compared with the traditional sorting SCL/CA-SCL algorithm, IPS has little performance loss and low complexity. To conclude, the innovations of the proposed IPS are as follows:

- We propose a framework that uses permutation entropy to measure the complexity of path selection. By comparing different path extension methods, the best path extension method can be determined. Treating path selection as a binary classification problem brings us new solutions.
- We propose an intelligent path-selection method consisting of a neural network, a threshold, and a matching strategy, which reduces the latency of path selection. The simpler the network, the less the hardware resource complexity and latency.

The remainder of paper is as follows. Section 2 gives a short introduction to polar codes and LLR-based SCL/CA-SCL. Section 3 introduces the permutation entropy and the differences between various path extension schemes. Section 4 shows the design of IPS, the neural network, and the matching strategy. Section 5 provides the simulation results, and the conclusions are in Section 6.

## 2. Preliminaries

### 2.1. Polar Codes

A polar code P(N,K) of length *N* with *K* information bits is constructed by applying a linear transformation to the message word $u_{1}^{N}=\left\{u_{1},u_{2},\ldots ,u_{N}\right\}$ as

(1) $$x_{1}^{N}=u_{1}^{N}F^{⊗n},F=\left[\begin{array}{cc} 1 & 0\\ 1 & 1 \end{array}\right],$$

where $x_{1}^{N}=\left\{x_{1},x_{2},\ldots ,x_{N}\right\}$ is the codeword, $F^{⊗n}$ is the *n*-th Kronecker power of the polarizing matrix F, and $n=\log_{2}N$. The message word $u_{1}^{N}$ contains a set A of *K* information bits and a set F of N−K frozen bits. In this paper, frozen bits are selected according to the 5GNR standard. Binary phase-shift keying (BPSK) modulation and an additive white Gaussian noise (AWGN) channel model are considered.

(2) $$y_{1}^{N}=\left(1-2x_{1}^{N}\right)+z,$$

where **1** is an all-one vector with size *N*, and $z\in R^{N}$ is the AWGN noise vector with variance $\sigma^{2}$ and a zero mean.

In addition, for an $\left(N,K_{I}\right)$ CRC-polar concatenated code, the inner code is an (N,K) polar code, and the outer code is a $\left(K,K_{I}\right)$ CRC code. The CRC-concatenated polar code rate is $R=K_{I}/N$, and the set of information bits is A, |A|=K. The message word $b_{1}^{K_{I}}=\left\{b_{1},b_{2},\ldots ,b_{K_{I}}\right\}$ is encoded as the CRC encoded codeword $c_{1}^{K}$:

(3) $$c_{1}^{K}=b_{1}^{K_{I}}G_{c},$$

where $G_{c}$ is the generator matrix generated by the CRC polynomial g(x). $K_{P}=K-K_{I}$ is the length of the CRC check bit.

Insert the CRC codeword $c_{1}^{K}$ into the information sequence $u_{1}^{N}$ according to the information bits set A, and obtain the codeword $x_{1}^{N}$ after polar encoding.

In the log-likelihood ratio (LLR) domain, the LLR vector $\left\{L_{1}^{\left(i\right)},i=1,2,\ldots ,N\right\}$ of the transmitted codeword is

(4) $$L_{1}^{\left(i\right)}=\ln\left(\frac{W(y_{i}|0)}{W(y_{i}|1)}\right)=\frac{2y_{i}}{\sigma^{2}}$$

### 2.2. LLR-Based Successive Cancellation List Decoding

The SC decoding algorithm can be regarded as a greedy search on the decoding tree. In each decision of the information bit, only the one with the larger posterior probability is selected. Obviously, once a bit error occurs in the decoding process, the decoding of the codeword fails. The SCL decoding algorithm is an enhanced version of the SC algorithm that includes a list of candidate paths of size *L*. In other words, the SCL decoding algorithm is a breadth-first algorithm on the decoding tree.

The SCL decoding algorithm can be divided into three stages. (1) Extend the candidate path until the candidate path size is *L*. (2) Extend the candidate paths to 2L; sort by 2L path metrics; select the most reliable *L* candidate paths. (3) The last information bit outputs the most reliable candidate path. In this paper, we discuss how to extend the candidate paths and how to select the most reliable *L* candidate paths.

In the implementation of the high-throughput CA-SCL decoder, LLR-based and approximate calculations are usually used to reduce complexity. LLRs are defined as

(5) $$L_{N}^{\left(i\right)}\left(y_{1}^{N},{\hat{u}}_{1}^{i-1}\right)=\ln\left(\frac{W_{N}^{\left(i\right)}\left(y_{1}^{N},{\hat{u}}_{1}^{i-1}|0\right)}{W_{N}^{\left(i\right)}\left(y_{1}^{N},{\hat{u}}_{1}^{i-1}|1\right)}\right).$$

The estimation ${\hat{u}}_{i}$ of information bits $u_{i}$ is defined as

(6) $${\hat{u}}_{i}=\left\{\begin{array}{ll} 0 & i\notin A,\\ \delta (L_{N}^{\left(i\right)}) & i\in A. \end{array}\right.$$

where $\delta \left(x\right)=\frac{1}{2}\left(1-sign\left(x\right)\right)$.

The LLR update rule is

(7) $$L_{N}^{\left(2i-1\right)}\left(y_{1}^{N},{\hat{u}}_{1}^{2i-2}\right)=f\left(L_{N/2}^{\left(i\right)}\left(y_{1}^{N/2},{\hat{u}}_{1,o}^{2i-2}⊕{\hat{u}}_{1,e}^{2i-2}\right),L_{N/2}^{\left(i\right)}\left(y_{N/2+1}^{N},{\hat{u}}_{1,e}^{2i-2}\right)\right),\;L_{N}^{\left(2i\right)}\left(y_{1}^{N},{\hat{u}}_{1}^{2i-1}\right)=g\left(L_{N/2}^{\left(i\right)}\left(y_{1}^{N/2},{\hat{u}}_{1,o}^{2i-2}⊕{\hat{u}}_{1,e}^{2i-2}\right),L_{N/2}^{\left(i\right)}\left(y_{N/2+1}^{N},{\hat{u}}_{1,e}^{2i-2}\right),{\hat{u}}_{2i-1}\right),$$

where the *g* function and the approximate *f* function are

(8) $$\begin{array}{ccc} f(a,b) & =sign(a)\cdot sign(b)\cdot min\{|a|,|b|\}, & \\ g(a,b,u_{s}) & ={\left(-1\right)}^{u_{s}}\cdot a+b. \end{array}$$

The path metric (PM) for the *i*-th bit of path *l* is defined as

(9) $${\mathrm{PM}}_{l}^{\left(i\right)}=\left\{\begin{array}{ccc} {\mathrm{PM}}_{l}^{\left(i-1\right)} & \; & \mathrm{if}\;{\hat{u}}_{i}\left[l\right]=\delta \left(L_{N}^{\left(i\right)}\left[l\right]\right),\\ {\mathrm{PM}}_{l}^{\left(i-1\right)}+\left|L_{N}^{\left(i\right)}\left[l\right]\right| & \; & \mathrm{otherwise}. \end{array}\right.$$

## 3. Path Selection and Permutation Entropy

In this section, we analyze the process of path selection in the SCL algorithm and establish a relationship with the permutation entropy.

### 3.1. Rethinking Path Selection

Among the *L* candidate paths maintained by the SCL algorithm, each path corresponds to a PM to represent the reliability of the path. In this paper, the smaller the PM, the more reliable the candidate path.

Assume that the PM metric of the (i−1)-th bit is $\left\{{\mathrm{PM}}_{l}^{\left(i-1\right)},l=1,2,\ldots ,L,i\in A\right\}$, After path extension, the PM extension (PME) values are $\left\{{\mathrm{PME}}_{l}^{\left(i-1\right)},l=1,2,\ldots ,2L,i\in A\right\}$. To find the most reliable *L* paths, the PME values are usually sorted to get $\left\{{{\mathrm{PME}}^{\prime }}_{l}^{\left(i-1\right)},l=1,2,\ldots ,2L,i\in A\right\}$, which satisfies ${{\mathrm{PME}}^{\prime }}_{1}^{\left(i-1\right)}\leq {{\mathrm{PME}}^{\prime }}_{2}^{\left(i-1\right)}\leq \ldots \leq {{\mathrm{PME}}^{\prime }}_{2L}^{\left(i-1\right)}$. Keep the *L* smallest PME values as the new metric values: $\left\{{\mathrm{PM}}_{l}^{\left(i\right)}={{\mathrm{PME}}^{\prime }}_{l}^{\left(i-1\right)},l=1,2,\ldots ,L,i\in A\right\}$.

For software simulations, we generally do not care how sorting is done. However, hardware resource consumption and delay are crucial aspects that must be taken into account while designing hardware.

For the expansion from $\left\{{\mathrm{PM}}_{l}^{\left(i-1\right)},l=1,2,\ldots ,L,i\in A\right\}$ to $\left\{{\mathrm{PME}}_{l}^{\left(i-1\right)},l=1,2,\ldots ,2L,i\in A\right\}$, we discuss the following extension schemes.

**Extension Scheme 1:** Extend path *l* to 2l−1 and 2l, where the hard decision of 2l−1 is zero and the hard decision of 2l is one.

(10) $${\mathrm{PME}}_{2l-1}^{\left(i-1\right)}=\left\{\begin{array}{ccc} {\mathrm{PM}}_{l}^{\left(i-1\right)} & \; & \mathrm{if}\;\delta (L_{N}^{\left(i\right)}\left[l\right])=0,\\ {\mathrm{PM}}_{l}^{\left(i-1\right)}+\left|L_{N}^{\left(i\right)}\left[l\right]\right| & \; & \mathrm{otherwise}. \end{array}\right.$$

(11) $${\mathrm{PME}}_{2l}^{\left(i-1\right)}=\left\{\begin{array}{cccc} \; & {\mathrm{PM}}_{l}^{\left(i-1\right)} & \; & \mathrm{if}\;\delta (L_{N}^{\left(i\right)}\left[l\right])=1,\\ \; & {\mathrm{PM}}_{l}^{\left(i-1\right)}+\left|L_{N}^{\left(i\right)}\left[l\right]\right| & \; & \mathrm{otherwise}. \end{array}\right.$$

In this way, it is easy to find the expanded original path after sorting.

**Extension Scheme 2:** Extend path *l* to 2l−1 and 2l, where the PM of 2l−1 is smaller than that of 2l.

(12) $$\begin{array}{c} {\mathrm{PME}}_{2l-1}^{\left(i-1\right)}={\mathrm{PM}}_{l}^{\left(i-1\right)},\\ {\mathrm{PME}}_{2l}^{\left(i-1\right)}={\mathrm{PM}}_{l}^{\left(i-1\right)}+\left|L_{N}^{\left(i\right)}\left[l\right]\right|. \end{array}$$

This is easy to extend, because 2l−1 always remains the same.

**Extension Scheme 3:** Extend path *l* to *l* and l+L, where the PM of *l* is smaller than l+L.

(13) $$\begin{array}{c} {\mathrm{PME}}_{l}^{\left(i-1\right)}={\mathrm{PM}}_{l}^{\left(i-1\right)},\\ {\mathrm{PME}}_{l+L}^{\left(i-1\right)}={\mathrm{PM}}_{l}^{\left(i-1\right)}+\left|L_{N}^{\left(i\right)}\left[l\right]\right|. \end{array}$$

In this case, the entire PME remains unchanged for l≤L.

In hardware implementation, schemes 2 and 3 are both considered. Due to the potential size relationship (${\mathrm{PME}}_{2l-1}^{\left(i-1\right)}<{\mathrm{PME}}_{2l}^{\left(i-1\right)}$ or ${\mathrm{PME}}_{l}^{\left(i-1\right)}<{\mathrm{PME}}_{l+L}^{\left(i-1\right)})$, the number of comparisons will be reduced.

Furthermore, not all PMEs require complete sorting. Partial path sorting simplifies the sorting operation and only needs to meet the condition of ${{\mathrm{PME}}^{\prime }}_{1}^{\left(i-1\right)}\leq {{\mathrm{PME}}^{\prime }}_{2}^{\left(i-1\right)}\leq \ldots \leq {{\mathrm{PME}}^{\prime }}_{L}^{\left(i-1\right)}<{{\mathrm{PME}}^{\prime }}_{m}^{\left(i-1\right)},m=L+1,L+2,\ldots ,2L$. This means that the number of comparisons can theoretically be reduced further.

In the next subsection, we explore a more idealized way of path selection by analyzing the permutation entropy.

### 3.2. Analysis Based on Permutation Entropy

Permutation entropy is used to describe the chaotic degree of a time series, which is calculated by the entropy based on the permutation patterns. A permutation pattern is defined as the order relationship among values of a time series.

#### 3.2.1. Definition of Permutation Entropy

We use permutation entropy to define the chaotic degree of a sequence ${\left\{x_{t}\right\}}_{t=1,\ldots ,T}$. a vector composed of the *n*-th subsequent values is constructed:

(14) $$s\mapsto \left(x_{s+1},x_{s+2},\ldots ,x_{x+n}\right).$$

*n* is the order of permutation entropy. The permutation can be defined as: $\pi =(r_{0}r_{1}\ldots r_{n-1})$, which satisfies

(15) $$x_{s+r_{0}}\leq x_{s+r_{1}}\leq \ldots \leq x_{s+r_{n-1}}$$

Obviously, there are a total of n! permutation patterns π. For each π, we determine the relative frequency (# means number):

(16) $$p\left(\pi \right)=\frac{\#\{s|0\leq s\leq T-n,\left(x_{s+1},\ldots ,x_{s+n}\right)\mathrm{has}\;\mathrm{type}\;\pi \}}{T-n+1}$$

**Definition** **1.** *The permutation entropy is defined as (n≥2):*

(17) $$PE\left(n\right)=-\sum_{i=1}^{n!}p\left(\pi_{i}\right)\log p\left(\pi_{i}\right).$$

Noting that PE∈[0,logn!], a normalized permutation entropy can be defined as:

(18) $$PE_{norm}\left(n\right)=-\frac{1}{\log n!}\sum_{i=1}^{n!}p\left(\pi_{i}\right)\log p\left(\pi_{i}\right).$$

#### 3.2.2. Permutation Entropy in Path Selection

In the SCL decoder, the size of the PM value is *L* and the size of the PM value after extension is 2L.

We assume that the original sequence $\left\{{\mathrm{PM}}_{l}^{\left(i-1\right)},l=1,2,\ldots ,L,i\in A\right\}$ before extension is unordered (order-3 permutation entropy for analysis):

(19) $$h_{0}=\sup PE\left(3\right)=\log\left(3!\right)=\log\left(6\right).$$

The $\left\{{\mathrm{PME}}_{l}^{\left(i-1\right)},l=1,1,\ldots ,2L,i\in A\right\}$ after **Extension Scheme 1** is obviously unordered:

(20) $$h_{1}=\sup PE\left(3\right)=\log\left(3!\right)=\log\left(6\right).$$

However, after **Extension Scheme 2**, the situation becomes different. Permutation π=(210) never appears. The maximum permutation entropy becomes

(21) $$h_{2}=\sup PE\left(3\right)=\log\left(5\right).$$

**Extension Scheme 3** is similar to **Extension Scheme 1** but more ordered than **Extension Scheme 1**:

(22) $$h_{3}=h_{1}.$$

**Figure 2:**
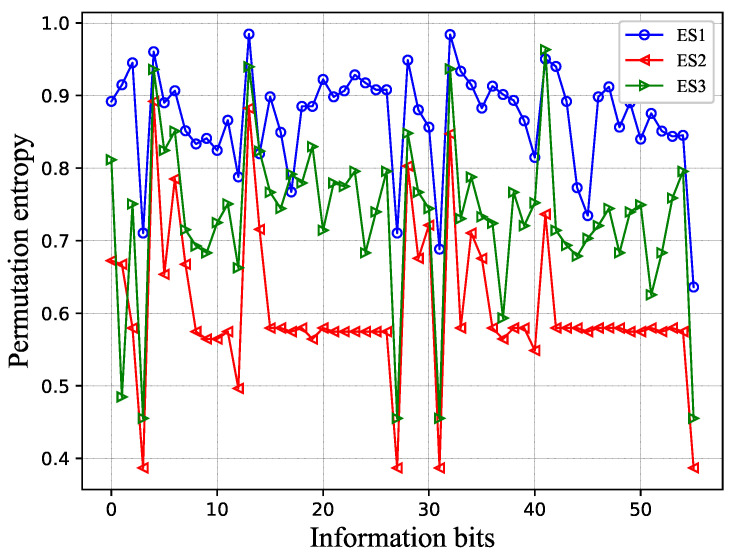
Permutation entropy of PME values in P(64,32)L=16 SCL decoder. ES1/2/3: **Extension Scheme 1/2/3**.

Figure 2 shows the PME permutation entropy under the same codeword and noise conditions. The horizontal axis has the information bits (only if fully extended to *L* paths), and the vertical axis has the order-3 permutation entropy. The logarithm is in base 2. From this result, we can find many interesting conclusions. (1) As in the previous analysis, in most cases, $PE{\left(3\right)}_{ES1}<PE{\left(3\right)}_{ES3}<PE{\left(3\right)}_{ES2}$. (2) Some specific information bits have high permutation entropy, and some specific information bits have low permutation entropy. This means that, for a specific set of information bits, sorting algorithms with different complexities can be used to reduce the overall complexity of the algorithm.

#### 3.2.3. Ideal-Path-Selection Method

The traditional sorting method, whether it involves complete sorting or a partial sorting, will get ${{\mathrm{PME}}^{\prime }}_{1}^{\left(i-1\right)}\leq {{\mathrm{PME}}^{\prime }}_{2}^{\left(i-1\right)}\leq \ldots \leq {{\mathrm{PME}}^{\prime }}_{L}^{\left(i-1\right)}<{{\mathrm{PME}}^{\prime }}_{m}^{\left(i-1\right)},m=L+1,L+2,\ldots ,2L$, which is ${\mathrm{PM}}_{1}^{\left(i\right)}\leq {\mathrm{PM}}_{2}^{\left(i\right)}\leq \ldots \leq {\mathrm{PM}}_{L}^{\left(i\right)},i\in A$. The new PM values are completely ordered:

(23) $$PE{\left(n\right)}_{Trad}^{\left(i\right)}=0.$$

However, the paths in the list do not actually need to be sorted. Due to the existence of noise, the PM value cannot fully represent the reliability of the path. Every surviving path has the potential to be the correct path. This can be reflected in the CA-SCL algorithm, which selects the path that passes the CRC check instead of the path with the smallest PM. Thus, it is not necessary to sort the surviving path every time, and this leads to the following corollary.

**Corollary** **1.** *The ideal path selection can be viewed as a binary classification, where all paths are classified as more reliable and less reliable. Assuming that the $\left\{{\mathrm{PME}}_{l}^{\left(i-1\right)},l=1,2,\ldots ,2L,i\in A\right\}$ goes through the ideal path selection, then it should satisfy*

(24) $${{\mathrm{PME}}^{\prime }}_{m}^{\left(i-1\right)}<{{\mathrm{PME}}^{\prime }}_{n}^{\left(i-1\right)},\forall 1\leq m\leq L,L+1\leq n\leq 2L.$$

*Keep the L smallest PME values as the new metric value $\left\{{\mathrm{PM}}_{l}^{\left(i\right)}={{\mathrm{PME}}^{\prime }}_{l}^{\left(i-1\right)},l=1,2,\ldots ,L,i\in A\right\}$; the permutation entropy of PM should satisfy*

(25) $$PE{\left(n\right)}_{Ideal}^{\left(i\right)}\geq 0.$$

**Figure 3:**
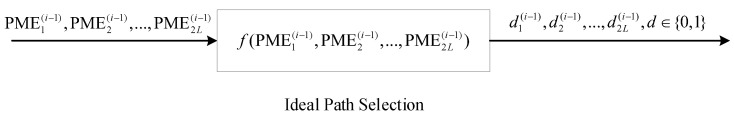
Ideal-path-selection system model.

The ideal path selection can be represented using the system model of Figure 3, where the input is $\left\{{\mathrm{PME}}_{l}^{\left(i-1\right)},l=1,2,\ldots ,2L,i\in A\right\}$ and the output is $\left\{d_{l}^{\left(i-1\right)},l=1,2,\ldots ,2L,d\in \left\{0,1\right\}\right\}$. $d_{l}^{\left(i-1\right)}$ is the label of the PME; $d_{l}^{\left(i-1\right)}=1$ means this is a more reliable path and needs to be kept. On the contrary, $d_{l}^{\left(i-1\right)}=0$ means discard it.

Before the extension, the path metric has the same complexity, $PE{\left(n\right)}^{\left(i-1\right)}$. After different path selections, different permutation entropies $PE{\left(n\right)}^{\left(i\right)}$ are obtained. Obviously, the larger $PE{\left(n\right)}^{\left(i\right)}$, the lower the complexity of path selection.

Finding such a function is almost impossible, but luckily, we can approximate this function using neural network methods. In the next section, we go into the details of the design and use of the neural network.

## 4. Intelligent-Path-Selection-Aided Decoding Algorithm

In this section, we design a general path-selection neural network. We describe the overall IPS structure in the first subsection and explain each detail in the following subsections.

### 4.1. Intelligent-Path-Selection-Aided Decoder’s Structure

The intelligent path selection input is 2L PMEs, and the output is *L* PMs. We designed the following intelligent path-selection architecture to accomplish these functions.

**Figure 4:**
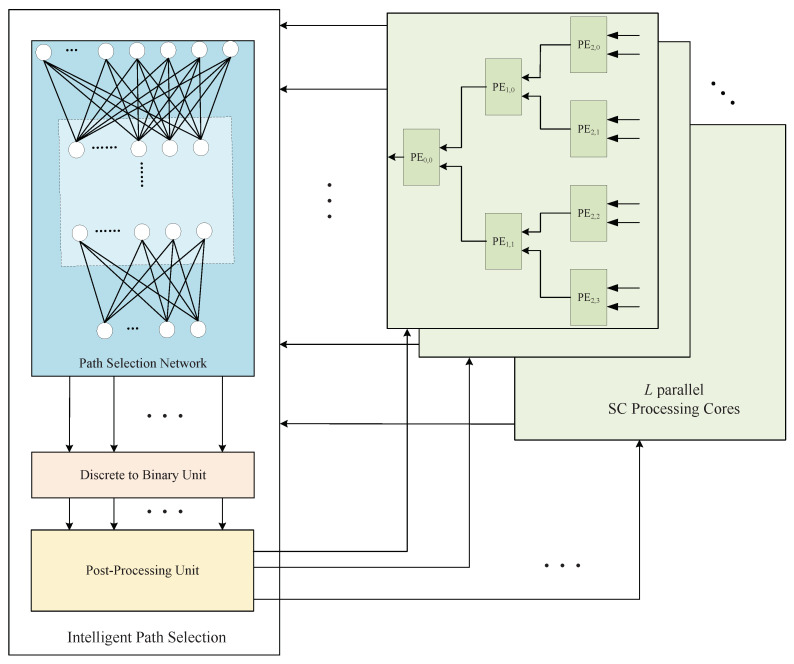
IPS-aided decoder’s structure.

As shown in Figure 4, the input PMEs can be recalculated by the network to obtain the new path reliability metrics $\left\{o_{l}^{\left(i-1\right)},l=1,2,\ldots ,2L,o\in \left(0,1\right)\right\}$. The larger $o_{l}^{\left(i-1\right)}$ is, the more reliable the path. Next, a threshold divides the paths into good paths ($d_{l}^{\left(i-1\right)}=1$) and bad paths ($d_{l}^{\left(i-1\right)}=0$). Up till now, we have completed the binary classification process and picked out the most reliable paths. However, there is the small drawback that the number of the most reliable paths is not always equal to *L*, which sometimes results in wasted resources. Thus, we designed a post-processing unit such that the number of IPS outputs is always *L*.

It is worth noting that training does not need to be done online. After the network has been trained, the parameters are put into the SCL/CA-SCL decoder. Therefore, the complexity of the training does not affect the latency of the decoding. However, complex networks are not conducive to hardware implementation. Thus, for the network structure, the simpler the better.

### 4.2. Path-Selection Neural Network

#### 4.2.1. Data Preparation

For input vector $\left\{{\mathrm{PME}}_{l},l=1,2,\ldots ,2L\right\}$, 2L PME values are sorted ${{\mathrm{PME}}^{\prime }}_{1}<{{\mathrm{PME}}^{\prime }}_{2}<\ldots <{{\mathrm{PME}}^{\prime }}_{2L}$. If ${\mathrm{PME}}_{l}\leq {{\mathrm{PME}}^{\prime }}_{L}$, label vector is defined as $d_{l}=1$; else, $d_{l}=0$. Thus, the sum of label vector $d=\{d_{l},l=1,2,\ldots ,2L\}$ is *L*.

#### 4.2.2. Neural Network Model

**Figure 5:**
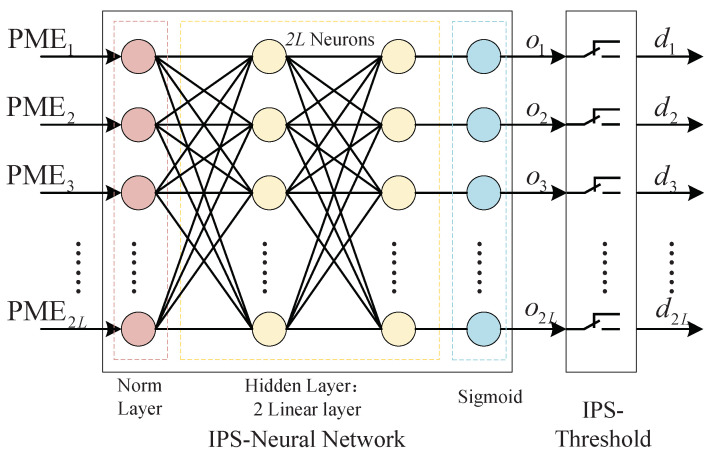
IPS neural network model.

We use a simple neural network as the basic component of IPS (IPS-NN), including a normalization layer, two linear layers, and a sigmoid layer. Each layer of the network has 2L neurons. Figure 5 shows the IPS neural network model.

The binary cross entropy (BCE) is considered as the loss function:

(26) $$BCE=-\frac{1}{2L}\sum_{l=1}^{2L}\left[d_{l}\log\left(o_{l}\right)+\left(1-d_{l}\right)\log\left(1-o_{l}\right)\right],$$

where $o_{l}$ is *l*-th output of the IPS-NN.

The IPS threshold is a simple switch structure used to convert the network’s output discrete value o into a binary value $\hat{d}$.

(27) $${\hat{d}}_{l}=\left\{\begin{array}{ccc} 1 & \; & o_{l}>T,\\ 0 & \; & o_{l}\leq T, \end{array}\right.l=1,2,\ldots ,2L,$$

where *T* is the threshold value. Obviously, ${\hat{d}}_{l}=1$ means that the metric value of this path is small, and it is a potential successful decoding path (good path); otherwise, it is a bad path.

#### 4.2.3. IPS-NN Configuration and Results

We trained IPS-NN with N=64,R=1/2,L=16. The detailed hyperparameters for training are shown in Table 1.

**Definition** **2.** *The accuracy of the neural network is defined as:*

(28) $$Acc=\frac{\sum_{S}\sum_{l=0}^{2L}{𝟙}_{\left\{{\hat{d}}_{l}=d_{l}\right\}}}{2L\cdot |S|},$$

*S is test data set and |S| is the size of the test data set. $\hat{d}$ is the IPS output vector. ${𝟙}_{\left\{\cdot \right\}}$ is an indicator function that takes value one if the argument is true and zero otherwise. We consider each element in the vector as the standard of accuracy, rather than the entire vector. Even if the output vector is not exactly equal to the label, as long as the correct path is included, this vector is valid for the SCL algorithm.*

The IPS-NN network was implemented using the Pytorch framework. For the test set, the IPS-NN achieved an accuracy of 98.3%. However, this accuracy rate cannot completely determine the performance of the entire decoder. What is important is the performance of the entire CA-SCL decoder after replacing metric sorting with IPS.

### 4.3. Post-Processing Unit

In the previous section, we proposed IPS-NN and IPS-Threshold. It is worth noting that the output value of IPS is $\hat{d}$. We expect the sum of $\hat{d}$ to be *L*, but in fact, the sum of $\hat{d}$ is related to the network output and threshold. We need to perform post-processing operations on the network’s output.

**Figure 6:**
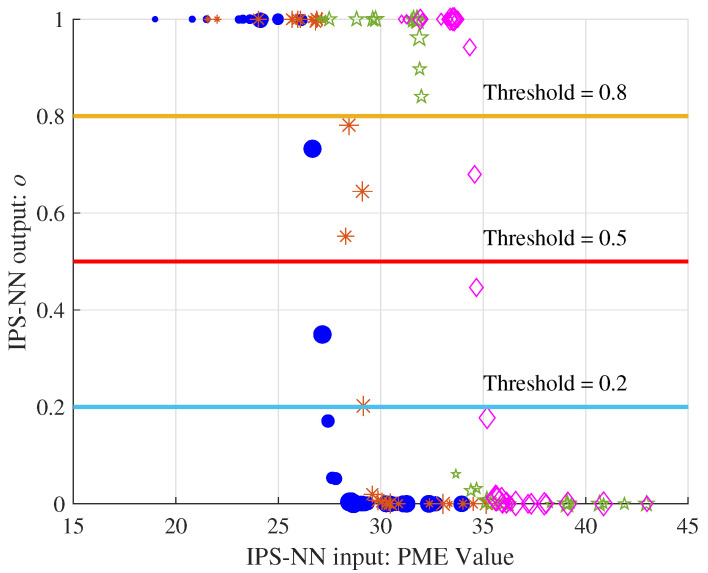
The output of the four sets of PME through the IPS-NN.

Figure 6 shows four sets of PME values selected in the decoding process with N=256,R=1/2,L=16. The size of the shape represents the order the input PME value. After IPS-NN, four sets of output values in the range (0,1) are obtained.

We can observe two phenomena: (1) The same PME value has different outputs in different sets, which shows that the IPS-NN can well adapt to the PME value of the entire set. In decoding the different bits, the PM value continues to increase, and the network is applicable. (2) The number of IPS output ${\hat{d}}_{l}=1$ is related to the threshold value; the larger the threshold, the smaller the output ${\hat{d}}_{l}=1$.

We denote the sum of ${\hat{d}}_{l}$ as Ω. In order to solve the effect of the threshold on the output results, there are two solutions: (1) Using a variable threshold, use the dichotomy method to find the threshold value of when Ω≠L. (2) Add a compensation strategy to make the decoder output *L* paths when Ω≠L. Method 1 has good performance but increases the complexity of each decoding stage. Method 2 has some performance losses, but it is not sensitive to the decoding threshold and can have a larger threshold range. In this paper, we use method 2 and propose a simple matching strategy to make the decoder work smoothly. The matching strategy is intended to be compatible with conventional SCL/CA-SCL decoders and does not need to provide additional performance. Our network chooses the best paths, even if they are smaller than the size of the list. However, there is only one correct path, and it is most likely in the path we have chosen.

**Strategy 1.(Discard Matching Strategy)**: When Ω≠L, a simple discarding matching strategy is adopted. If Ω>L, discard some good path; if Ω<L, discard some decoding path. A detailed description of the discard matching strategy is given in Algorithm 1.

If Ω>L, we simply discard the good paths larger than *L*. If Ω<L, we supplement the unselected paths at the front of the list with the candidate paths. Obviously, the performance of this strategy is related to the extension scheme. We can set a larger threshold to ensure Ω<L in most cases, and the performance of the matching strategy is determined by the extension scheme.

In addition to using the discard matching strategy, different strategies, such as random selection, can also be used. These strategies will affect the performance of the decoder to a certain extent.

**Algorithm 1** Discard matching strategy.**Input:** Current 2L extended path $\left\{{\mathrm{PME}}_{l}^{\left(i-1\right)},l=1,2,\ldots ,2L\right\}$, IPS output $\hat{d}$ **Output:**
*L* survival paths PM $\left\{{\mathrm{PM}}_{l}^{i},l=1,2,\ldots ,L\right\}$ 1: cnt_pm=1 2: **for **
s=1,2,…,2L
**do** 3:    **if** ${\hat{d}}_{l}=1\;\mathrm{and}\;cnt\_pm\leq L$ **then** 4:     ${\mathrm{PM}}_{cnt\_pm}^{i}={\mathrm{PME}}_{s}^{\left(i-1\right)}$ 5:      cnt_pm=cnt_pm+1 6:    **end if** 7: **end for** 8: **if **
Ω<L
**then** 9:    **for** s=1,2,…,2L **do** 10:      **if** ${\hat{d}}_{l}=0\;\mathrm{and}\;cnt\_pm\leq L$ **then** 11:         ${\mathrm{PM}}_{cnt\_pm}^{i}={\mathrm{PME}}_{s}^{\left(i-1\right)}$ 12:         cnt_pm=cnt_pm+1 13:      **end if** 14:    **end for** 15: **end if**


### 4.4. Hardware Design for IPS-NN

In this subsection, we provide a basic parallel NN structure for more convenient representation of latency. In the simulation, the norm layer was not necessary, and its removal does not affect performance. As for the sigmoid function, it can be implemented using ROM as a look-up table. The key to the latency is the design of the hidden layer.

The design of the hidden layers is based on two basic principles. (1) Parallelism: the nodes of the same layer depend only on the previous layer and can be computed simultaneously. (2) Pipelined operation, as each hidden layer depends only on the output of the previous layer; therefore, hardware resources can be reused to achieve pipelined operation. Therefore, we only need to design for one hidden layer node.

**Figure 7:**
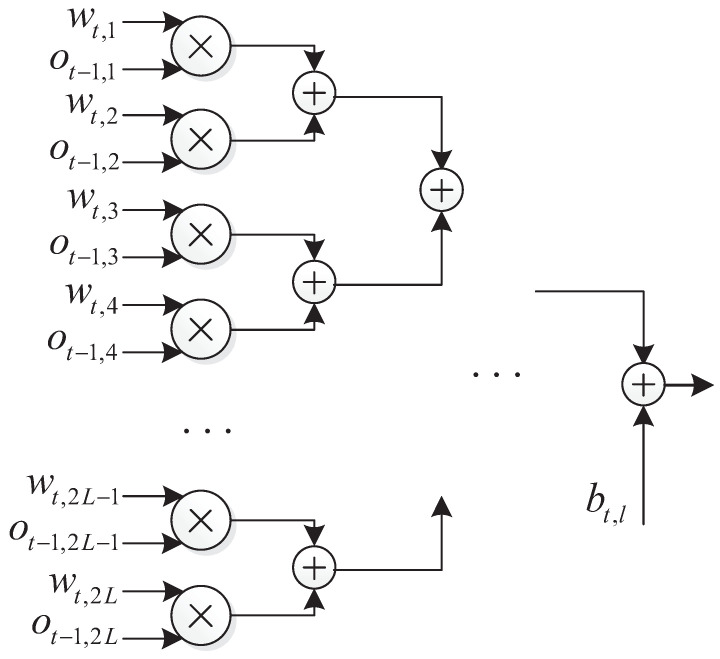
Structure of the *l*-th node of the *t*-th layer.

The output of the *l*-th node at the *t*-th hidden layer is

(29) $$o_{t,l}=f_{a}\left(\sum_{m=1}^{2L}\left(w_{t,m}\cdot o_{t-1,m}\right)+b_{t,l}\right),$$

where $f_{a}\left(\right)$ is the activation function, which can be implemented using a look-up table. One parallel structure of this hidden nodes is as Figure 7.

In the structure, multiplication can be performed in parallel, and the time consumed by addition is $\log_{2}\left(2L\right)$. Thus, the time consumption for a single hidden layer is

(30) $$D_{hidden}=1+\log_{2}\left(2L\right)=2+\log_{2}\left(L\right).$$

It is worth noting that, unlike the compare-and-swap (CAS) used for the traditional sorter, the NN implementation relies on adders and multipliers. With the same structure, the data-bit width also affects the throughput of the hardware.

## 5. Simulation Results

We put the IPS trained in Section 4 into the SCL/CA-SCL decoder. Note that the *L* PMs of the SCL algorithm using IPS are unordered. Hence, it is necessary to sort from the last bit in order to output the path with the smallest PM. CA-SCL does not require any additional operations.

### 5.1. IPS and Extension Scheme Performance Analysis

**Figure 8:**
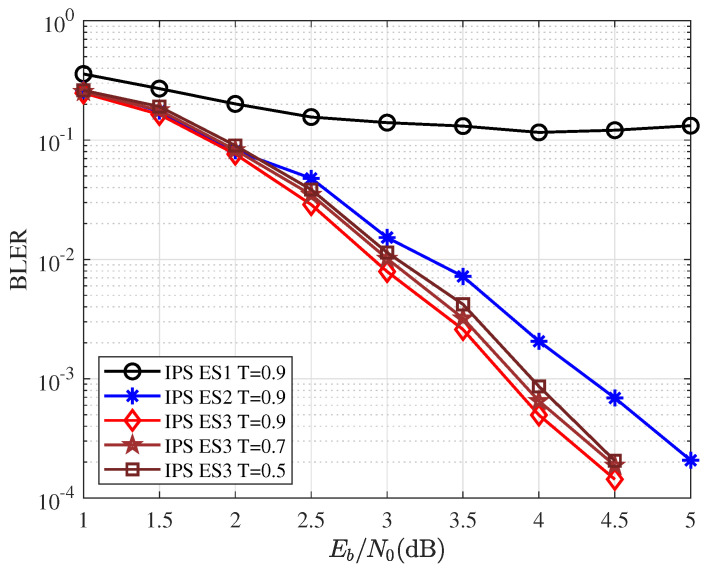
Same network, different extension scheme BLER comparison.

The training data for IPS were generated by P(64,32)L=16 following **Extension Scheme 2**. *T* was the threshold value. Given the codelengths N=64 and K=32, CRC length $K_{P}=6$. Figure 8 shows the block-error-ratio (BLER) performance for different extension schemes and thresholds. With the same threshold value T=0.9, **Extension Scheme 1** has very poor performance. The reason is that the original sequence has a large sorting entropy, so IPS cannot classify well, and the matching strategy does not make up for this deficiency. **Extension Scheme 3** has the best performance, mainly due to the matching strategy. Performance with different thresholds varies, and larger thresholds perform better.

### 5.2. SCL/CA-SCL Performance Comparison

**Figure 9:**
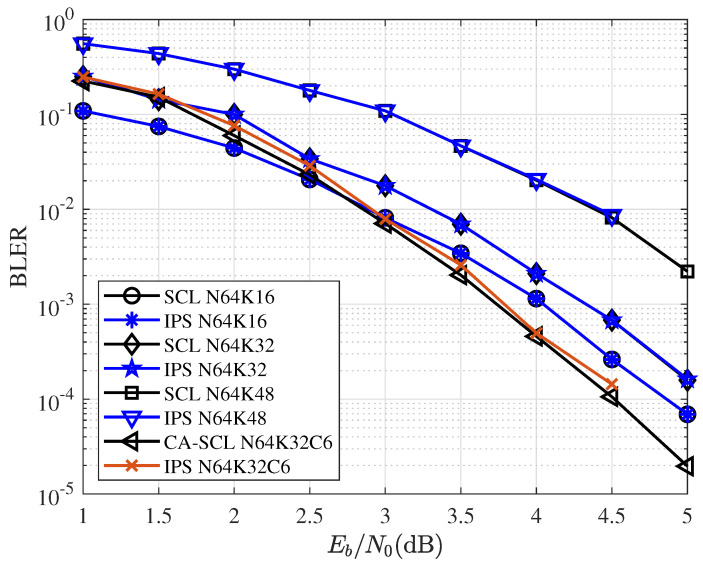
BLER performance comparisons for SCL/CA-SCL and IPS with block length N=64 and different code rates.

**Figure 10:**
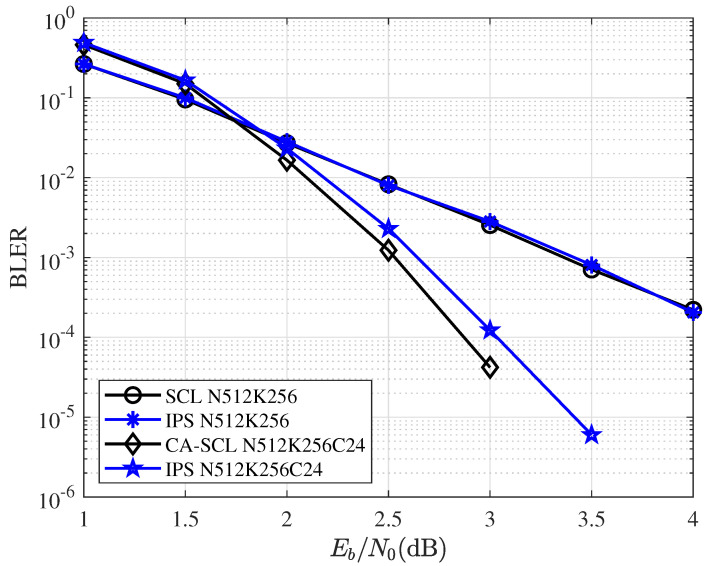
BLER performance comparisons for SCL/CA-SCL and IPS with block length N=512 and R=1/2.

Figure 9 and Figure 10 give the BLER performance comparisons for various coding and decoding schemes. CA-SCL uses 6-bit CRC with codelength N=64, and its generator polynomial is $g\left(x\right)=x^{6}+x^{4}+1$. CA-SCL uses 24-bit CRC with codelength N=512, and its generator polynomial is $g\left(x\right)=x^{24}+x^{23}+x^{21}+x^{20}+x^{17}+x^{15}+x^{13}+x^{12}+x^{8}+x^{4}+x^{2}+x+1$. As the figures show, the curves of SCL and IPS overlap. Under this configuration, IPS can also achieve the performance very close to that of CA-SCL. Additionally, for IPS decoding, the network was trained by P(64,32)L=16. The network trained with the same P(64,32)L=16 used for all different code lengths and code rates. Training for different code lengths and rates can further improve the performance of IPS.

### 5.3. Latency of Decoding

To evaluate the latency of IPS, we compare the theoretical delay with that of the state-of-the-art hybrid sorter (HS) [18] in the SCL algorithm and that of the local sorter [11] in the list Viterbi algorithm (LVA). The decoding delay of SCL/CA-SCL is divided into two parts. The first part is the decoding delay of SC. For unoptimized IPS and HS, this part of the delay is the same, 2(N−1). The second part is path selection. In the LVA algorithm, the same path selection is required. Therefore, we only compare the delay of a single path-selection operation.

The delay of the HS is

(31) $$T_{HS}=\frac{1}{2}\log_{2}\left(L\right)\cdot \left(1+\log_{2}\left(L\right)\right).$$

The IPS delay with *k* hidden layers is

(32) $$T_{IPS}=k\cdot \left(2+\log_{2}\left(L\right)\right).$$

The delay of the LVA local sorter is dependent on dividing the total list of size *L* into $2^{m}$ small lists ($L^{\prime }=L/2^{m}$):

(33) $$T_{LVA}=\frac{1}{2}\left(1+\log_{2}\frac{L}{2^{m}}\right)\left(2+\log_{2}\frac{L}{2^{m}}\right)-\log_{2}\frac{L}{2^{m}},$$

the last term of the formula is due to the fact that the local sorter does not sort with a metric. This means that the local sorter just pick the best path at each state node on the trellis.

**Figure 11:**
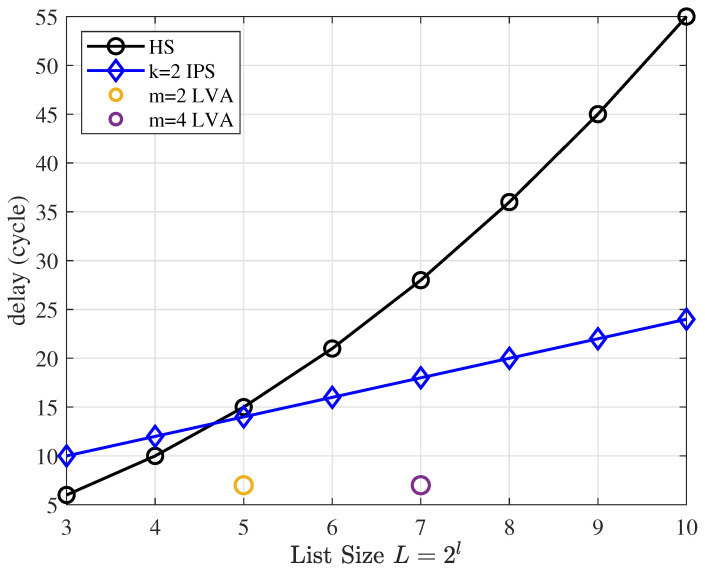
Single path-selection operation latency comparison.

As shown in Figure 11, compared to HS, IPS has lower latency for large lists, but the increase in speed is much lower than for HS. A local sorter is a special algorithm that only sorts at each state nodes on the trellis of the Viterbi algorithm, which results in a large list being split into smaller lists, reducing complexity. Meanwhile, a similar idea is used in IPS: sorting between surviving paths is not necessary, so the local sorter has lower latency than a small-list sorter.

## 6. Conclusions

In this paper, we proposed a new path-selection method. We first analyzed the permutation entropy of the path metric. With the help of neural networks, we proposed an approximation scheme for ideal path selection named IPS. Compared with traditional solutions, IPS showed little performance loss and lower theoretical latency. We believe the proposed path-selection method is helpful for building a low-latency SCL decoder.

## Figures and Tables

**Table 1:** Hyperparameters used for training the IPS-NN.

Parameter	Value
Optimizer	SGD
Learning rate	0.01
Training SNR (Eb/N0)	1dB, 100,000 frames
training sets:test sets	3:1
batch size	64
**Accuracy**	0.9836425

## Data Availability

Not applicable.

## Abbreviations

The following abbreviations are used in this manuscript:

SCL	Successive Cancellation List
CA-SCL	CRC-Aided Successive Cancellation List
IPS	Intelligent Path Selection
PM/PME	Path Metric/Path Metric Extension
